# Effectiveness of a Smoking Cessation Intervention for Methadone-Maintained Women: A Comparison of Pregnant and Parenting Women

**DOI:** 10.1155/2011/567056

**Published:** 2011-07-28

**Authors:** Amber M. Holbrook, Karol A. Kaltenbach

**Affiliations:** MATER, Department of Pediatrics, Jefferson Medical College, Thomas Jefferson University, 1233 Locust Street, Suite 401, Philadelphia, PA 19107, USA

## Abstract

Women in substance abuse programs have high rates of smoking. Pregnancy represents a unique opportunity for intervention, but few data exist to guide tailoring of effective interventions. In this study, 44 pregnant and 47 nonpregnant opioid-dependent women enrolled in comprehensive substance abuse treatment received a 6-week smoking cessation intervention based on the 5A's counseling model. The number of daily cigarettes decreased by 49% for pregnant patients and 32% for nonpregnant patients at the 3-month followup. Length of time in substance abuse treatment did not correlate with smoking cessation or reduction for either group. Factors predicting reduction of cigarette smoking differed for pregnant versus nonpregnant patients. For pregnant patients, lower levels of nicotine use prior to intervention and self-reported cigarette cravings predicted successful reduction in smoking. For nonpregnant patients, lower affiliative attachment to cigarettes, reliance on cigarettes for cognitive enhancement, and greater sense of control predicted more successful outcomes.

## 1. Introduction

Tobacco use represents a significant long-term risk to women's health [1, 2], and cigarette smoking is currently one of the leading preventable causes of poor pregnancy outcomes, as well as infant mortality and morbidity [3]. Smoking during pregnancy is associated with increased risk of preterm birth [4–6], placental abruption, placenta previa, low birth weight [6–8], and sudden infant death syndrome (SIDS) [9, 10]. The risks related to cigarette smoking continue following birth with child exposure to second hand tobacco smoke associated with an increased incidence of respiratory ailments such as asthma, respiratory infections, and bronchiolitis [11–13]. 

Estimates of the prevalence of smoking in participants in methadone maintenance programs range from 85 to 98% [14–17]. However, few substance abuse treatment programs offer smoking cessation interventions, and smoking cessation is often viewed as a low priority by treatment program staff [18]. Treatment staff may perceive substance-dependent individuals as possessing low motivation for smoking cessation [19] or believe that engaging in efforts to reduce nicotine dependence may be overwhelming for substance-dependent individuals, particularly those early in their recovery [18]. Some studies have also suggested that methadone maintenance may compound difficulty quitting smoking with a dose-dependent effect on tobacco craving and nicotine withdrawal symptoms [20, 21] affected through increased rate of nicotine metabolism or alteration to the sensitivity of nicotine receptors [22]. The reinforcing effects of nicotine, such as enhancement of cognitive performance, may also be stronger in methadone-maintained individuals [23]. 

Fortunately, pregnancy often represents a unique motivation for smoking cessation among women who use nicotine [24], and substance-dependent individuals frequently self-report a desire to quit smoking [25]. However, efforts to encourage smoking cessation in methadone maintenance programs have had limited success, with reduction in smoking appearing to be a more realistic goal than cessation [26]. Recently it has been suggested that smoking cessation interventions for pregnant women may need to be tailored to their specific needs, similar to intervention for dependence or abuse of other substances [27]. However, little data exist to suggest what factors are associated with effectiveness of smoking cessation interventions in pregnant women, or how these may differ from nonpregnant women in substance abuse treatment programs. 

This study compares outcomes of a 6-week smoking cessation intervention for pregnant and nonpregnant methadone-maintained women enrolled in a comprehensive outpatient substance abuse treatment program. The effectiveness of the intervention is compared between the two groups, and factors associated with successfully reducing or eliminating cigarette use in pregnant and nonpregnant women are examined.

## 2. Methods

### 2.1. Participants

Participants were 44 opioid-dependent pregnant women and 47 opioid-dependent parenting women receiving comprehensive outpatient substance abuse treatment. Patients who were identified by their primary counselors as nicotine dependent were referred for a six-week group smoking cessation intervention based on the 5 A's counseling model [28]. The 5 A's counseling approach is a five-step intervention that has been proven effective for use with pregnant women and is consistent with strategies developed by the National Cancer Institute and American Medical Association [29]. The steps include: asking about tobacco use; advising to quit; assessing patient motivation to quit; assisting in quit attempt; arranging for followup. Group content included assessment of current nicotine use, education on risks of tobacco use and benefits of cessation, identification of patient motivations to quit and triggers to smoke, and coping skills. Groups ran from April 2009 to March 2010 and included 8–15 patients per group.

### 2.2. Procedures

The Timeline Follow Back (TLFB) was administered during the week prior to the intervention. At the initial administration, pregnant participants were asked to report on their cigarette use from one month prior to pregnancy to the present. Parenting patients were asked to report on their cigarette use for one month prior to the intervention. The TLFB was administered again at the group intervention's midpoint (3 weeks), at the conclusion of the six-week group, and then once monthly for three months postintervention. An anonymous 10-item group evaluation was also administered at the close of the six-week group. The evaluation assessed patient knowledge of the risks of tobacco use, skills for managing cravings, and satisfaction with the content and format of the group. The Wisconsin Inventory of Smoking Dependence Motives (WISDM) was also administered to all patient one week prior to the intervention and again at the 3-month followup.

### 2.3. Measures

#### 2.3.1. Timeline Follow Back (TLFB)

The TLFB is a calendar format measure that utilizes memory aids such as important dates and events to aid recall of substance use over a specified period of up to 12 months. It has been used for over thirty years in clinical and research settings [30].

#### 2.3.2. Wisconsin Inventory of Smoking Dependence Motives (WISDM)

The WISDM is a 68-item measure of smoking dependence to assess the underlying motivations for smoking [31]. It is based on the theory that multiple motives for tobacco use comprise the construct of tobacco dependence, not solely nicotine dependence. These motives may contribute to tobacco use, withdrawal, and relapse. It has 13 subscales with internal consistency estimates ranging from 0.78 to 0.89 [32]. Subscales are Affiliative Attachment, Automaticity, Behavioral Choice-Melioration, Cognitive Enhancement, Craving, Cue Exposure, Loss of Control, Negative Reinforcement, Positive Reinforcement, Social-Environmental Goads, Taste and Sensory Properties, Tolerance, and Weight Control. Participants are asked to rate each of the 68 items on a scale of 1–7 with 1 being “not at all true of me” and 7 being “extremely true of me”.

#### 2.3.3. Group Evaluation

A ten-item anonymous questionnaire assessed increase in patient knowledge regarding risks of cigarette smoking, benefits of cessation, and response to cravings; perceived support for smoking cessation; readiness to change; satisfaction with group format and facilitator. Participants used a 5-point Likert scale to respond to items such as “I learned ways to control my cravings for cigarettes”, “The program taught me things I did know before about smoking”, and “I believe I am given the support I need to refrain from smoking”.

### 2.4. Data Analysis

Descriptive statistics were obtained for all variables initially included in the model. *t*-tests were conducted for key variables (age at entry to substance abuse treatment, length of time in substance abuse treatment, number of daily cigarettes at the beginning of intervention, percent decrease in smoking reporting in months prior to intervention) to determine if significant differences existed between the pregnant and nonpregnant groups prior to intervention. 

Four analyses were conducted utilizing multiple ordinary least squares regression. Outcome variables were the percent change in number of cigarettes smoked from beginning of the intervention to one month postintervention (PC1), and three months postintervention (PC3), for both the pregnant and nonpregnant groups. Stepwise regression was conducted to select variables for the final models. Initial predictor variables included maternal age, methadone dose, time in substance abuse treatment at start of the smoking cessation intervention (TimeTx), average number of cigarettes smoked daily prior to intervention (Cig Prior), percent change in number of cigarettes smoked daily from one month prior to intervention to week 1 of intervention (PC Base-Wk1), number of daily cigarettes smoked at week 1 of the intervention (Cigs Wk1), urine drug analysis positive for illicit or nonprescribed substances during the 6-week smoking intervention, satisfaction with the intervention as measured by total scores on the group evaluation (Satisfaction), and the 13 subscales of the WISDM. Six variables (PC base-Wk1, Cigs Wk1, Satisfaction, and three subscales of the WISDM: Loss of Control, Craving, Automaticity) were retained in the two models for pregnant patients, and five variables (five subscales of the WISDM: Affiliative Attachment, Cognitive Enhancement, Taste and Sensory Properties, Loss of Control, Automaticity) were retained in the two models for the nonpregnant women.

A multiple analysis of variance (MANOVA) was conducted to determine between group differences for pregnant versus nonpregnant patients on three variables: percent change in cigarette smoking from beginning of the intervention to the one-month followup (PC1), percent change from beginning of the intervention to the 3-month followup (PC3), and group satisfaction (Satisfaction).

## 3. Results

Descriptive statistics for pregnant and nonpregnant patients are presented in Table 1. No significant differences existed between the two groups on maternal age (26.7 yrs versus 27.8 yrs; *P* = 0.9) or methadone dose (143.6 mg versus 128.7 mg; *P* = 0.2). The overall sample was predominantly Caucasian, with a higher percentage of African-American and Latina patients in the nonpregnant group (Table 1). Length of time in substance abuse treatment (Time Tx) was significantly longer for the nonpregnant patients (35.1 wks versus 192.0 wks; *P* = 0.0001) (Table 1). 

Pregnant patients reported a significantly higher number of daily cigarettes prior to the intervention (18.8 versus 13.2; *P* = 0.003) (Table 2). However, no significance between group differences was found in number of cigarettes smoked at week 1 of the intervention, suggesting that pregnant women significantly decreased their rates of smoking prior to intervention. Mean number of daily cigarettes reported was not statistically significant between the pregnant and nonpregnant groups at the end (week 6) of the intervention, or at the 1-month or 3-month followups. However, mean number of cigarettes decreased over time for both groups and continued to decline during the 3-month postintervention followup (Table 2). Average number of daily cigarettes for the pregnant group decreased by 49% from week 1 of the intervention to the 3-month followup. For nonpregnant patients, mean number of daily cigarettes decreased by 32%.

### 3.1. Multivariate Regression

Following model selection utilizing stepwise regression, six variables were retained in the model to predict outcomes at the 1-month and 3-month followups for pregnant women. A greater percent decrease in number of daily cigarettes smoked prior the start of the intervention (PC Base-Wk1) (*b* = −0.12; *P* = 0.01), as well as a higher number of daily cigarettes reported at the beginning of the intervention (Cigs Wk1) (*b* = −1.33; *P* = 0.001) were both associated with smaller decreases in number of cigarettes at the 1-month followup for pregnant patients (Table 3). Greater patient satisfaction with the intervention correlated with smaller decreases in number of cigarettes (*b* = −12.6; *P* = 0.05). Higher scores on the WISDM subscale craving were also associated with a smaller decrease in cigarette smoking at the 1-month followup (*b* = −2.13; *P* = 0.03) with subscales Automaticity and Loss of Control showing trends toward significance (Table 3). Only patient satisfaction with the intervention predicted number of daily cigarettes at the 3-month followup (*b* = −69.35; *P* = 0.04). 

For the nonpregnant patients, five subscales of the WISDM were retained in the model to predict 1-month and 3-month outcomes. Only the Affiliative Attachment (*b* = −1.21; *P* = 0.02) and Cognitive Enhancement (*b* = −1.67; *P* = 0.003) predicted percent change in daily cigarettes at the 1-month followup (PC1), with higher scores on these WISDM subscales correlating with smaller PC1 (Table 4). The Taste and Sensory Properties subscale also showed a trend toward significance (*b* = −1.09; *P* = 0.06). Loss of Control and Automaticity were not significant in the final model predicting outcomes at the 1-month followup. Cognitive Enhancement remained a significant predictor for percent change in daily cigarettes for nonpregnant patients at the 3-month followup (PC3) (*b* = −2.17; *P* = 0.01). Higher scores on the Loss of Control subscale also predicted smaller decreases in PC3 (*b* = −2.02; *P* = 0.05). Affiliative Attachment, Taste and Sensory Properties, and Automaticity subscales all showed trends toward a negative impact on PC3, but none reached significance at the 0.05 level (Table 4).

### 3.2. MANOVA

MANOVA results indicate that there were no significant differences between pregnant and nonpregnant groups on the percent decrease in number of daily cigarettes from baseline at the 1-month (PC1) (*f* = 0.18; *P* = 0.67) or 3-month followups (PC3) (*f* = 0.03; *P* = 0.87). No significant differences were found between groups on satisfaction with the intervention (*f* = 1.46; *P* = 0.21).

## 4. Conclusions

While only a small percentage of patients in either the pregnant or nonpregnant groups ceased nicotine use entirely, reductions in number of cigarettes smoked daily were substantial for many patients, and these reductions were maintained, or even increased, over time. The number of people who quit smoking entirely increased from the end of the intervention to the 3-month followup, and the mean number of daily cigarettes decreased following the intervention for both groups as well, suggesting that it may take time to integrate new information and implement the coping skills learned in the group. 

No significant differences were found in the number of cigarettes smoked at the 1-month or 3-month followups, or in the percent decrease of daily cigarettes from the beginning of the intervention to the followups between groups, suggesting that the intervention was equally efficacious for both pregnant and nonpregnant women. However, pregnant patients reported a greater self-imposed decrease in cigarette smoking leading up to the intervention. While both the pregnant and nonpregnant patient groups were similar in demographics and level of nicotine dependence prior to the intervention, the pregnant patients had also been in substance abuse treatment significantly fewer weeks than nonpregnant patients. This is expected since the discovery of pregnancy is a common motivator for entry into substance abuse treatment. The decrease in cigarette smoking prior to intervention may represent the effects of entry into substance abuse treatment, or the motivation of discovery of pregnancy on nicotine use behaviors.

Although outcomes were similar for pregnant and non-pregnant patients, different factors predicted successful outcomes for the two groups. Number of cigarettes smoked daily at the start of the intervention, as well as the decrease the individual was able to affect prior to intervention influenced outcomes for pregnant women. This is consistent with previous literature suggesting that the heavier nicotine use correlates with less successful outcomes in smoking cessation efforts [33, 34]. However, for pregnant women, level of craving for cigarettes appeared to be a significant factor, whereas for nonpregnant women, the affiliative attachment to cigarettes and their role in cognitive enhancement appeared to be more central. Perhaps the most puzzling finding is that those pregnant women who were more satisfied with the intervention demonstrated smaller decreases in smoking at followup. It is possible that these women represented patients in the precontemplation stage who appreciated receiving the information contained in the intervention but were not ready to commit to cessation of nicotine use.

**Limitations**

Conclusions are limited by reliance on self-report data, which is subject to recall bias and socially desirable responding. Patients may underestimate, inaccurately recall, or wish to downplay the number of cigarettes smoked, particularly pregnant patients. Further research on the effectiveness of smoking cessation interventions may benefit from use of additional biological measure, such as cotinine testing, to evaluate reports of reductions in or cessation of nicotine use.

This study is also limited by small sample size. Lack of power may have obscured relationships which would have reached significance had the n been greater. In particular, subscales of the WISDM showed trends towards predicting decreases in number of daily cigarettes at the 1-month and 3-month followups but did not reach significance in the final models.

**Implications for Clinical Practice**

This study provides evidence that substantial reductions in cigarette smoking are possible for methadone-maintained patients, even following a relatively brief intervention. It also suggests that smoking cessation interventions may be effective even for patients that are relatively early in their recovery from substance abuse. However, as it appears that reductions occurred over time and required integration of the new skills, longer smoking cessation interventions or follow-up support may be useful in maintaining or increasing gains over time.

Results also indicate that the factors associated with success of smoking cessation interventions may differ for pregnant versus nonpregnant women. For pregnant women, pregnancy represents a unique motivation to decrease their nicotine use, and many pregnant patients appear to have begun this process prior to intervention and were able to further decrease their use following intervention. Heavier smoking as well as greater self-imposed decrease in cigarette smoking prior to intervention appeared to lessen the effectiveness of the intervention for pregnant women, consistent with previous literature. Higher scores on the craving subscale of the WISDM suggest that for pregnant women, particularly those who are newer to substance abuse treatment, increased focus on the management of cravings may be especially helpful. Further research is needed on efficacious treatment for nicotine dependence in this special population; however, this study does provide evidence that smoking cessation interventions are a worthwhile endeavor for women in substance abuse treatment programs.

## Figures and Tables

**Table 1:** Descriptive statistics total sample.

	Pregnant	Nonpregnant	*P*
	Mean/percent (SD)	Mean/percent (SD)	
Maternal age	26.7 (5.0)	27.8 (6.0)	0.9
Race/ethnicity			
Caucasian	97.7%	87.5%	
African-American	2.3%	10.4%	
Latina	0%	2.1%	
Time in treatment (wks)	35.1 (5.6)	192.0 (22.7)	**0.0001**
Methadone dose (mgs)	143.6 (48.6)	128.7 (67.5)	0.2

**Table 2:** Average number of daily cigarettes by time point.

Time	Pregnant	Nonpregnant	
	Mean (SD)	Mean (SD)	*P*
Prior to intervention	18.8 (10.1)	13.2 (1.0)	**0.003**
Week 1 intervention	15.2 (13.8)	12.4 (1.1)	0.2
Week 6 (end intervention)	8.3 (6.5)	9.3 (5.6)	0.4
1-month followup	8.3 (5.9)	8.6 (5.9)	0.8
3-month followup	7.9 (7.5)	8.5 (6.4)	0.7

**Table 3:** Correlates of decrease in smoking at followup: pregnant patients.

	1-month			3-month		
	*b*	*t*	*P*	*b*	*t*	*P*
PC Base-Wk 1	−0.12	−2.68	**0.01**	−2.52	−1.39	0.17
Cigs Wk 1	−1.33	−3.50	**0.001**	−2.26	−1.73	0.09
Satisfaction	−12.60	−2.03	**0.05**	−69.35	−2.19	**0.04**
Automaticity	−1.29	−1.87	0.07	−1.26	−0.56	0.58
Craving	−2.13	−2.22	**0.03**	−2.53	−0.81	0.43
Loss of control	−1.61	−1.85	0.07	−1.34	−0.48	0.64

*PC Base-Wk1: percent change in daily cigarettes prior to intervention; Cigs Wk1: mean daily cigarettes at start of intervention; Satisfaction: satisfaction with intervention; WISDM subscales: Automaticity, Craving, Loss of control.

**Table 4:** Correlates of decrease in smoking at followup: nonpregnant patients.

	1-month			3-month		
	*b*	*t*	*P*	*b*	*t*	*P*
Affiliative attachment	−1.21	−2.49	**0.02**	−1.42	−1.91	0.06
Cognitive enhancement	−1.67	−3.10	**0.003**	−2.17	−2.61	**0.01**
Taste and sensory properties	−1.09	−1.93	0.06	−1.53	−1.83	0.08
Automaticity	−0.66	−1.15	0.26	−1.51	−1.77	0.09
Loss of control	−0.98	−1.24	0.22	−2.20	−2.02	**0.05**

*WISDM subscales: Affiliative attachment, Cognitive enhancement, Taste and sensory properties, Automaticity, Loss of control.

## References

[1] Ransohoff DF, Chin MH, Blow FC (2006). National institutes of health state-of-the-science conference statement: tobacco use: prevention, cessation, and control. *Annals of Internal Medicine*.

[2] Center for Disease Control and Prevention (2002). Women and smoking: a report of the surgeon general. *Morbidity and Mortality Weekly Report*.

[3] Crawford JT, Tolosa JE, Goldenberg RL (2008). Smoking cessation in pregnancy: why, how, and what next. *Clinical Obstetrics and Gynecology*.

[4] Burguet A, Kaminski M, Abraham-Lerat L (2004). The complex relationship between smoking in pregnancy and very preterm delivery. Results of the Epipage study. *International Journal of Obstetrics and Gynaecology*.

[5] Kolås T, Nakling J, Salvesen A (2000). Smoking during pregnancy increases the risk of preterm births among parous women. *Acta Obstetricia et Gynecologica Scandinavica*.

[6] Hammoud AO, Bujold E, Sorokin Y (2005). Smoking in pregnancy revisited: findings from a large population-based study. *American Journal of Obstetrics and Gynecology*.

[7] Bernstein IM, Mongeon JA, Badger GJ, Solomon L, Heil SH, Higgins ST (2005). Maternal smoking and its association with birth weight. *Obstetrics and Gynecology*.

[8] Steyn K, De Wet T, Saloojee Y, Nel H, Yach D (2006). The influence of maternal cigarette smoking, snuff use and passive smoking on pregnancy outcomes: the birth to ten study. *Paediatric and Perinatal Epidemiology*.

[9] Cnattingius S (2004). The epidemiology of smoking during pregnancy: smoking prevalence, maternal characteristics, and pregnancy outcomes. *Nicotine and Tobacco Research*.

[10] U.S. Department of Health and Human Services (2001). *Women and Smoking: A Report of the Surgeon General*.

[11] Tutka P, Wielosz M, Zatoński W (2002). Exposure to environmental tobacco smoke and children health. *International Journal of Occupational Medicine and Environmental Health*.

[12] Jaakkola JJK, Gissler M (2004). Maternal smoking in pregnancy, fetal development, and childhood asthma. *American Journal of Public Health*.

[13] US Department of Health and Human Services (2006). *The Health Consequences of Involuntary Exposure to Tobacco Smoke: A Report of the Surgeon General*.

[14] Best D, Lehmann P, Gossop M, Harris J, Noble A, Strang J (1998). Eating too little, smoking and drinking too much: wider lifestyle problems among methadone maintenance patients. *Addiction Research and Theory*.

[15] Richter KP, Gibson CA, Ahluwalia JS, Schmelzle KH (2001). Tobacco use and quit attempts among methadone maintenance clients. *American Journal of Public Health*.

[16] Clarke JG, Stein MD, McGarry KA, Gogineni A (2001). Interest in smoking cessation among injection drug users. *American Journal on Addictions*.

[17] Shadel WG, Stein MD, Anderson BJ (2005). Correlates of motivation to quit smoking in methadone-maintained smokers enrolled in a smoking cessation trial. *Addictive Behaviors*.

[18] Fuller BE, Guydish J, Tsoh J (2007). Attitudes toward the integration of smoking cessation treatment into drug abuse clinics. *Journal of Substance Abuse Treatment*.

[19] Guydish J, Passalacqua E, Tajima B, Manser ST (2007). Staff smoking and other barriers to nicotine dependence intervention in addiction treatment settings: a review. *Journal of Psychoactive Drugs*.

[20] Schmitz JM, Grabowski J, Rhoades H (1994). The effects of high and low doses of methadone on cigarette smoking. *Drug and Alcohol Dependence*.

[21] Stark MJ, Campbell BK (1993). Cigarette smoking and methadone dose levels. *American Journal of Drug and Alcohol Abuse*.

[22] Spiga R, Schmitz J, Day J (1998). Effects of nicotine on methadone self-administration in humans. *Drug and Alcohol Dependence*.

[23] Hayaki J, Stein MD, Lassor JA, Herman DS, Anderson BJ (2005). Adversity among drug users: relationship to impulsivity. *Drug and Alcohol Dependence*.

[24] Lumley J, Oliver S, Chamberlain C, Oakley L (2009). Interventions for promoting smoking cessation during pregnancy. *Cochrane Database of Systematic Reviews*.

[25] Chisolm MS, Brigham EP, Lookatch SJ, Tuten M, Strain EC, Jones HE (2010). Cigarette smoking knowledge, attitudes, and practices of patients and staff at a perinatal substance abuse treatment center. *Journal of Substance Abuse Treatment*.

[26] Stein MD, Weinstock MC, Herman DS, Anderson BJ, Anthony JL, Niaura R (2006). A smoking cessation intervention for the methadone-maintained. *Addiction*.

[27] Heil SH, Linares Scott T, Higgins ST (2009). An overview of principles of effective treatment of substance use disorders and their potential application to pregnant cigarette smokers. *Drug and Alcohol Dependence*.

[28] Fiore MC, Jaen CR, Baker TB (2008). *Treating Tobacco Use and Dependence: 2008 Update. Clinical Practice Guideline*.

[29] The American College of Obstetricians and Gynecologists (ACOG) (2002). *Smoking Cessation during Pregnancy: A Clinician’s Guide to Helping Pregnant Women Quit Smoking*.

[30] Sobell LC, Sobell MB, American Psychiatric Association (2008). Alcohol timeline followback (TLFB). *Textbook of Psychiatric Measures*.

[31] Piper ME, Piasecki TM, Federman EB (2004). A multiple motives approach to tobacco dependence: the wisconsin inventory of smoking dependence motives (WISDM-68). *Journal of Consulting and Clinical Psychology*.

[32] Shenassa ED, Graham AL, Burdzovic JB, Buka SL (2009). Psychometric properties of the wisconsin inventory of smoking dependence motives (WISDM-68): a replication and extension. *Nicotine and Tobacco Research*.

[33] Colman GJ, Joyce T (2003). Trends in smoking before, during, and after pregnancy in ten states. *American Journal of Preventive Medicine*.

[34] Polańska K, Hanke W, Sobala W (2005). Smoking relapse one year after delivery among women who quit smoking during pregnancy. *International Journal of Occupational Medicine and Environmental Health*.

